# Biofilm formation by *Streptococcus agalactiae*: influence of environmental conditions and implicated virulence factors

**DOI:** 10.3389/fcimb.2015.00006

**Published:** 2015-02-04

**Authors:** Roberto Rosini, Immaculada Margarit

**Affiliations:** Novartis VaccinesSiena, Italy

**Keywords:** streptococcal infections, *Streptococcus agalactiae*, biofilms, group B streptococcus, pili/fimbriae/curli

## Abstract

*Streptococcus agalactiae* (Group B *Streptococcus*, GBS) is an important human pathogen that colonizes the urogenital and/or the lower gastro-intestinal tract of up to 40% of healthy women of reproductive age and is a leading cause of sepsis and meningitis in the neonates. GBS can also infect the elderly and immuno-compromised adults, and is responsible for mastitis in bovines. Like other Gram-positive bacteria, GBS can form biofilm-like three-dimensional structures that could enhance its ability to colonize and persist in the host. Biofilm formation by GBS has been investigated *in vitro* and appears tightly controlled by environmental conditions. Several adhesins have been shown to play a role in the formation of GBS biofilm-like structures, among which are the protein components of pili protruding outside the bacterial surface. Remarkably, antibodies directed against pilus proteins can prevent the formation of biofilms. The implications of biofilm formation in the context of GBS asymptomatic colonization and dissemination to cause invasive disease remain to be investigated in detail.

## Introduction

The beta-hemolytic Gram-positive *Streptococcus agalactiae* (Group B Streptococcus, GBS) is often encountered in the gastro-intestinal and the genital tract of healthy women as part of the normal flora. From this site, the bacteria can reach the newborn through the birth canal and cause sepsis and/or meningitis (Gibbs et al., 2004; Dando et al., 2014). GBS is also an important cause of morbidity and mortality in the elderly and in immuno-compromised adults. Primary manifestations of adult GBS disease include bacteremia, skin and soft tissue infections, pneumonia, osteomyelitis and urinary tract infections (Edwards et al., 2005; Skoff et al., 2009). GBS can also colonize the mammary gland of ruminants, where it is able to survive for long periods causing clinical and sub-clinical mastitis (Keefe, 1997).

GBS colonization and infection of target tissues requires the capacity of these bacteria to adhere and to persist in mucosal epithelial surfaces. In this habitat, the formation of biofilm-like communities could facilitate microbial survival and proliferation by enhancing resistance to host defenses and nutrient deprivation.

The present review summarizes recent studies investigating the capacity of GBS to form biofilm-like structures *in vitro*, how this mode of growth is affected by environmental conditions, and the contribution of adhesin virulence factors.

## Experimental evidence of GBS biofilm formation and influence of environmental conditions

Initial evidence suggesting that GBS could be implicated in the formation of biofilms came from studies by Donlan and Costerton where GBS bacteria were found on intrauterine devices in association with other known biofilm formers such as *Staphylococcus aureus* and *Staphylococcus epidermidis* (Donlan and Costerton, 2002).

Macroscopic assays were set up to investigate the biofilm forming capacity of GBS strains belonging to different lineages (Rinaudo et al., 2010). According to these type of assay, bacteria are cultured under static conditions in the wells of plastic tissue culture plates and, after several washes, microbial three-dimensional structures are stained with crystal violet or similar compounds (O'Toole et al., 2000).

More restrictive experimental methods have recently been set up to better discriminate between GBS weak and strong biofilm formers. One of these approaches mimics fluid circulation in the host by using flow conditions in laminar chamber systems (Konto-Ghiorghi et al., 2009). An alternative multiwell-based protocol is based on plate incubation under shaking and removal of non-attached bacteria by extensive washing, followed by replacement of the growth medium (D'Urzo et al., 2014).

Environmental conditions are known to strongly influence the capacity to form biofilm by many bacterial species (Froeliger and Fives-Taylor, 2001; Moscoso et al., 2006; Manetti et al., 2007). Several studies have investigated GBS *in vitro* biofilm formation using one of the above described methods and different growth media, with contrasting results. For instance, Kaur et al. (2009), Borges et al. (2012), and Yang et al. (2012) investigated biofilm production under neutral and acidic pH conditions. They found larger biofilm amounts at pH 6.5 compared to pH 4.2, probably due to poor bacterial growth at low pH. By contrast, Ho et al. (2013) found that low pH induced biofilm formation in nutrient-limited chemically defined medium (M9YE) and not in rich media like Todd-Hewitt Broth (THB). Konto-Ghiorghi et al. (2009) reported that a uniform biofilm was produced only on Luria Broth and RPMI 1640 supplemented with 1% glucose. The need for glucose for GBS biofilm formation was confirmed by Rinaudo et al. who also demonstrated that this sugar does not affect planktonic bacterial growth (Rinaudo et al., 2010). Previous studies had shown that in the GBS related species *Streptococcus pyogenes* the glucose biofilm enhancing effect was the direct result of acidification due to metabolic production of organic acids (Manetti et al., 2010). Evidence that acidic pH and not glucose concentration was the environmental signal driving GBS biofilm formation *in vitro* was obtained by D'Urzo et al. (2014). The authors tested a wide panel of strains in both buffered or non-buffered nutrient-rich (THB) and nutrient-limited (RPMI) media, in the presence or absence of glucose. Strong biofilm formation was observed only in glucose-containing non-buffered media and in low pH media even in the absence of glucose. In an *in vivo* setting, exposure of GBS to the acidic milieu of the vagina could be the signal sensed by the bacteria to grow in a sessile mode in this site. In this context, temporal shifts in GBS loads were recently observed in a mouse model of vaginal colonization (Carey et al., 2014), and have also been reported in humans (Hansen et al., 2004). It is tempting to speculate that pH variations and consequently GBS biofilm formation on epithelial cells could affect GBS carriage fluctuations.

Therefore, the discrepancies between the different studies in the observed capacity of GBS strains to form biofilm-like structures *in vitro* can possibly be explained by the use of different types of assays and growth conditions.

## Contribution of pili and other surface virulence factors to GBS biofilm formation

Long filamentous structures protruding from the surface of Gram-positive bacteria were discovered in the last decade (Ton-That and Schneewind, 2004; Soriani and Telford, 2010). These structures resemble the pili found in Gram-negative bacteria, although in Gram-positives pilus protein components are linked by covalent bonds.

**Figure 1:**
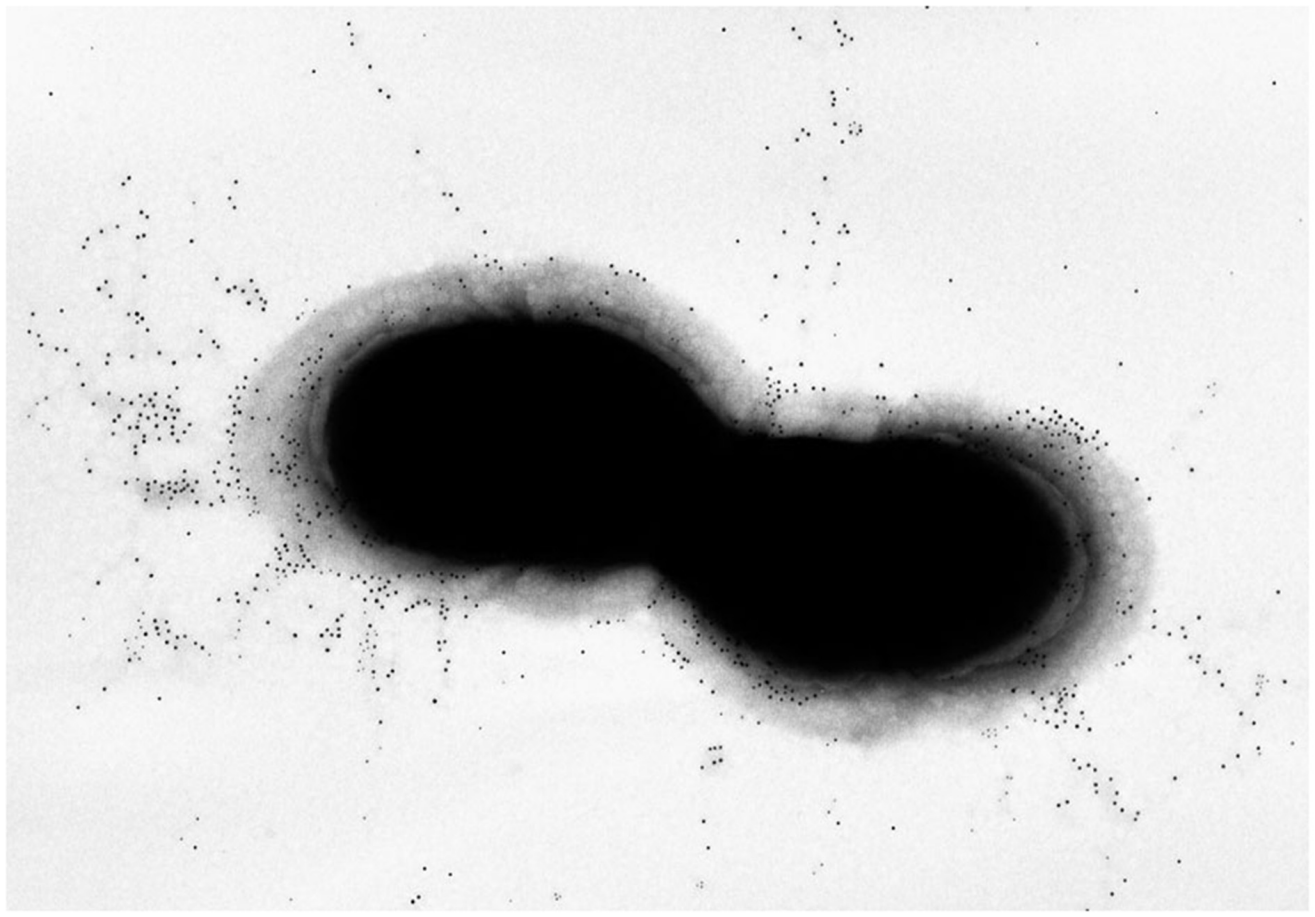
**Pilus Island-2a**. Immunogold transmission electron microscopy of GBS 515 grown to exponential phase. Bacteria were incubated with polyclonal sera raised against the corresponding pilus 2a backbone variant obtained as recombinant protein, and labeled with secondary antibodies conjugated with 10 nm gold nanoparticles.

Pili in *S. agalactiae* were discovered during screening of multiple genomes for surface-exposed protein antigens as possible vaccine targets (Lauer et al., 2005). By SDS-PAGE, the pilus polymers appeared as a ladder of bands ranging from 150 kDa to beyond the resolution of the gels, while immune electron microscopy revealed long appendages protruding outside the capsule that covers the bacterial surface (Figure 1). The genes encoding the GBS pilus machinery are clustered in three related genomic islands (Islands PI-1, -2a, and -2b) located in two separate loci flanked by direct repeats and conserved genes. All islands contain three genes encoding the pilus components, i.e., one backbone protein essential for pilus assembly (BP) and two accessory proteins (AP1 and AP2), plus two genes encoding sortase enzymes catalyzing the covalent linkage of the pilus proteins into long polymers (Dramsi et al., 2006; Rosini et al., 2006). The pilus proteins of PI-1 and PI-2b differ by very few amino acids, while PI-2a is more variable with seven alleles described both for the BP and the AP1 presenting sequence identities between 48–98% for BP and 87–98% for AP2. Remarkably, mouse immunization with BP-1, BP-2b, and AP1-2a conferred protection against infection with a large panel of virulent strains, and at least one of the three islands is present in all GBS (Margarit et al., 2009; Madzivhandila et al., 2011; Martins et al., 2013).

The discovery of pili in Gram-positive pathogens raised the question on the possible role of these highly surface-exposed structures in host colonization and infection. Pioneering evidence for their involvement in cell adhesion and biofilm formation was obtained in *S. pyogenes* (Manetti et al., 2007).

**Figure 2:**
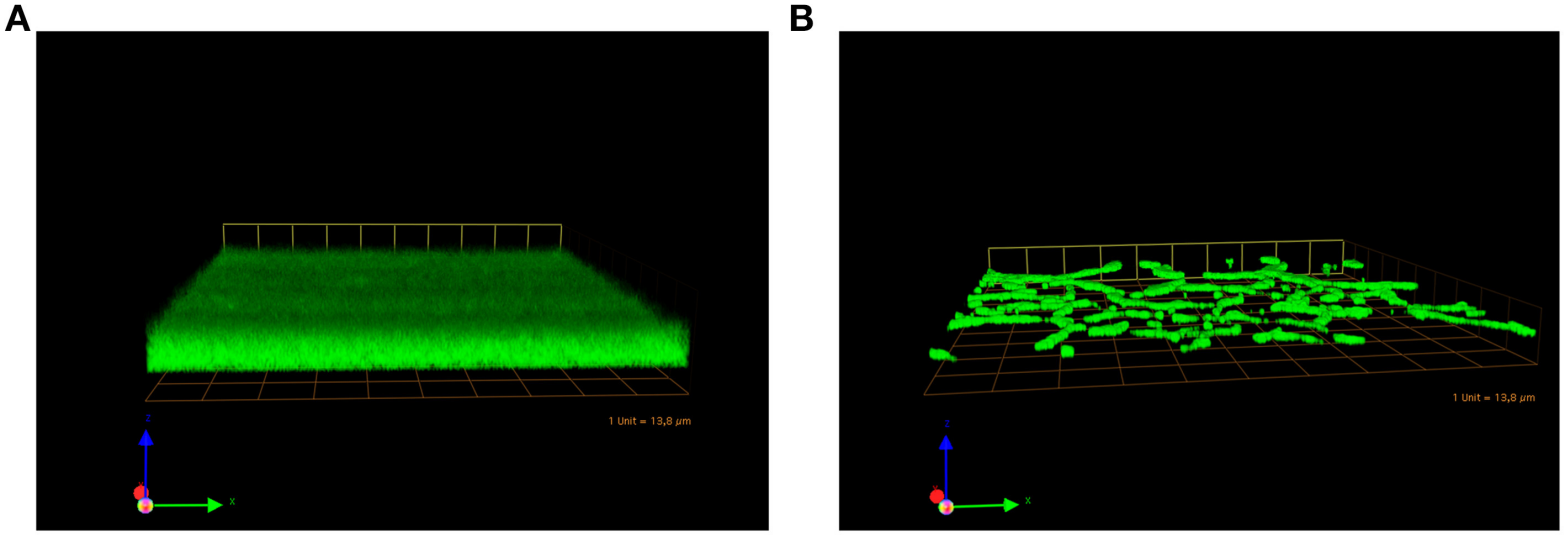
**Pilus Island-2a involvement in the formation of biofilms *in vitro***. Confocal scanning laser microscopy micrographs of biofilm development by GBS 515 **(A)** and its mutant derivative containing an in-frame deletion in the pilus backbone gene (Rosini et al., 2006) **(B)**. Bacteria were grown on glass coverslips under static conditions at 37°C for 72 h, fixed and stained with SYTO-9 prior to confocal analysis (magnification 60x).

Studies using GBS isogenic mutants lacking pilus 2a components or the sortase enzymes responsible for pilus polymerization and cell wall attachment, indicated a role of pili in host cell contact and in the formation of multi colony three-dimensional structures on abiotic surfaces (Konto-Ghiorghi et al., 2009; Rinaudo et al., 2010). These biofilm-like structures were also analyzed by confocal laser scanning microscopy where the wild-type GBS515 strain bearing pilus variant 2a and an isogenic mutant unable to assemble pili, were seeded on glass polylysine-coated coverslips and stained with a fluorescent dye. As shown in Figure 2, the wild-type strain formed structured multilayered aggregates of surface-adherent bacteria resembling a mature biofilm, while the deletion mutant did not.

Remarkably, antibodies directed against the backbone of pilus 2a and its main ancillary protein inhibited the formation of these biofilm-like structures in a dose dependent manner, while antibodies against the small ancillary protein located at the pilus base near the cell wall, did not show any effect (Rinaudo et al., 2010). The same study investigated biofilm formation by 289 GBS clinical isolates using the above described crystal violet assay under static conditions. A correlation between the high surface exposure of pilus 2a and the biofilm formation phenotype was observed.

In the more recent study by D'Urzo et al., the formation of biofilm-like structures *in vitro* by 389 GBS isolates was investigated under more stringent conditions to better discriminate between weak and strong biofilm formers (see above). Also in this case, a high variability among strains was observed both in pilus expression and in the capacity to form biofilms, even when they belonged to the same serotype or MLST phylogenetic lineage. A subset of serotype III strains belonging to the hyper virulent lineage ST-17 harboring both pilus 1 and 2b was shown to form stronger biofilms than all other tested strains, particularly at low pH (D'Urzo et al., 2014). Importantly, ST-17 strains are the most frequent cause of late-onset neonatal infections (Tazi et al., 2010). The proteins responsible for the higher capacity of this ST-17 subset of strains to form biofilms have not yet been identified, and could be possible targets to prevent colonization/disease of this hypervirulent lineage.

Park et al. investigated the phenotype of the CsrRS two-component regulatory system knockout, and showed an increase capacity of CsrRS mutant bacteria to adhere to host cells and to form biofilm-like structures on plate (Park et al., 2012). This regulatory effect of CsrRS on bacterial adherence and biofilm formation correlated with the expression of multiple surface adhesins but not of Pilus 1, excluding a role of this pilus variant in biofilm formation in the investigated isolate. The same and other authors identified BsaB/FbsC as a protein adhesin involved in biofilm formation and regulated by the CsrRS system (Buscetta et al., 2014; Jiang and Wessels, 2014); different from the pilus proteins, FbsC expression appeared slightly downregulated in a CsRS dependent manner at acidic versus neutral pH.

## Conclusions

Similar to other Gram-positive pathogens colonizing the human host, Group B *Streptococcus* can form multicellular communities that are expected to facilitate its persistence under environmental stress conditions. A number of *in vitro* studies have demonstrated that GBS forms biofilm-like structures on abiotic surfaces. Yet, the presence in these structures of the extracellular matrix typical of bacterial biofilms has not been investigated in this species. Additional studies are also needed to confirm the relevance of biofilm formation *in vivo*. If this would be the case, the involved virulence factors could constitute new therapeutic and preventive targets against this important human pathogen.

### Conflict of interest statement

Dr. Roberto Rosini and Dr. Immaculada Margarit are Novartis Vaccine employees. Dr. Immaculada Margarit holds Novartis stock options.
